# Burnout and Joy in the Profession of Critical Care Medicine

**DOI:** 10.1186/s13054-020-2784-z

**Published:** 2020-03-24

**Authors:** Meeta Prasad Kerlin, Joanne McPeake, Mark E. Mikkelsen

**Affiliations:** 1 Division of Pulmonary, Allergy, and Critical Care Medicine, Perelman School of Medicine at the University of Pennsylvania, Philadelphia, PA USA; 2 Palliative and Advanced Illness Research (PAIR) Center, Perelman School of Medicine at the University of Pennsylvania, Philadelphia, PA USA; 3 Center for Clinical Epidemiology and Biostatistics, Perelman School of Medicine at the University of Pennsylvania, Philadelphia, PA USA; 4 NHS Greater Glasgow and Clyde, Glasgow, UK; 5 University of Glasgow, School of Medicine, Dentistry and Nursing, Glasgow, UK

## Abstract

This article is one of ten reviews selected from the Annual Update in Intensive Care and Emergency Medicine 2020. Other selected articles can be found online at https://www.biomedcentral.com/collections/annualupdate2020. Further information about the Annual Update in Intensive Care and Emergency Medicine is available from http://www.springer.com/series/8901.

## Introduction

The intensive care unit (ICU) can be a stressful environment for patients and families, with well-established long-term consequences [1, 2]. The impact that this unique environment can have on healthcare professionals is being increasingly recognized [3–5]. Challenging ethical situations, exposure to high patient mortality and difficult daily workloads can lead to excessive stress for those caring for critically ill patients [3, 6, 7]. A growing body of literature suggests that this excessive stress and resultant moral distress can lead to burnout syndrome.

In this state-of-the-art review, we focus on the epidemiology of burnout syndrome in the ICU and the impact it can have on clinicians, patients, and the health service. Risk factors for burnout syndrome, alongside potential strategies to mitigate burnout and optimize fulfillment, will also be discussed.

### Burnout Syndrome

In 2016, the Critical Care Societies Collaborative, which includes the American Thoracic Society, the American Association of Critical Care Nurses, the American College of Chest Physicians, and the Society of Critical Care Medicine, convened a working group to focus attention on psychological health and well-being for providers of critical care. This official “Call for Action” statement defined burnout syndrome as an “individual response to particular work related events that manifest in people that do not have baseline psychological disorders” [3].

Burnout syndrome, described nearly half a century ago, is defined as a work-related condition characterized by three symptoms: emotional exhaustion, depersonalization, and a reduced sense of personal accomplishment [8, 9]. Burnout syndrome manifests when an individual’s perceived self-worth and expectations do not match those of the employers/organization [3, 4]. Although the concept of burnout syndrome, applied to healthcare providers, is still evolving and its causes and manifestations have overlap with other concepts such as compassion fatigue, for the purpose of clarity, this state-of-the-art review will focus on burnout and burnout syndrome.

**Fig. 1:**
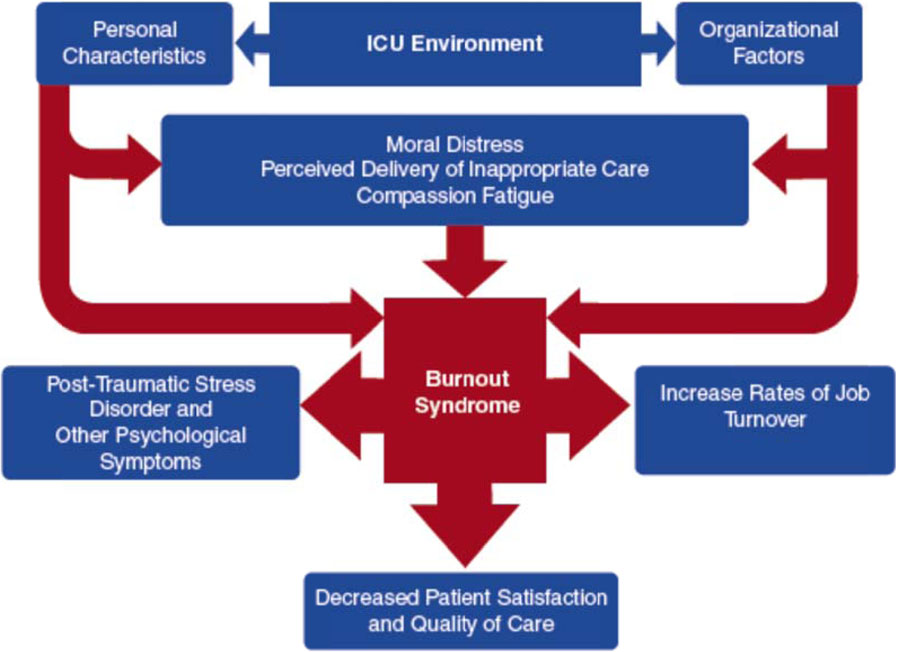
Risk factors associated with burnout syndrome and impact on the provider, care, and the healthcare system (Reprinted from [3] with permission of the American Thoracic Society. Copyright © 2019 American Thoracic Society)

In general, burnout manifests when one (or more) of six mismatches between individual and job is present: workload, control, reward, community, fairness, and values [9]. The six-mismatch framework has been applied to design interventions at the individual and organization level and, as described below, was simplified and applied to the profession of critical care medicine by the Critical Care Societies Collaborative (Fig. 1).

### Prevalence of Burnout Syndrome Among Critical Care Professionals

Multidisciplinary, coordinated care, delivered by caring and compassionate clinicians trained in critical care, is an essential component to high-quality critical care delivery [10]. Relative to other professions, burnout is more common among the “caring” professions [11], which partly explains the burnout epidemic present among healthcare professionals and critical care clinicians, in particular [3, 4, 10–18].

In cross-sectional studies, most critical care clinicians manifest one of the three classic features of burnout [3, 4]. For example, in a United States study of university hospital ICU nurses, 81% of critical care nurses experienced one or more symptoms of burnout [12], and severe burnout syndrome was found in 33% of critical care nurses and nursing assistants studied in a large French survey study [14].

Two large national surveys conducted more than a decade apart reveal the magnitude of what appears to be an enduring epidemic among critical care physicians. In a landmark, 1-day national survey conducted in 189 French ICUs in 2004, a high level of burnout was observed in 46.5% of critical care physicians [17]. In a survey of 15,069 United States physicians conducted in 2019, wherein critical care physicians comprised 1% of respondents, 44% of physicians surveyed were burned out, as were 44% of the critical care physicians surveyed [16]. Furthermore, 14% of survey respondents reported that they had thoughts of suicide. Burned out or depressed critical care physicians, who on average reported working longer hours, were less likely to seek professional help [16].

### Burnout and Fulfillment in Critical Care as a Profession

To date, the epidemiology of well-being among critical care professionals has focused on burnout assessed at a single time point. To gain a more complete understanding of critical care professional well-being, in line with the National Academy of Medicine recommendation to improve clinician well-being, which requires a commitment to “measure it, develop and implement interventions, and then remeasure it” [19], one health system implemented an initiative wherein they serially assess critical care provider well-being [18]. At each survey, section critical care physicians complete two, complementary, validated tools to measure burnout and professional fulfillment [18–21]. Notably, the initiative measures well-being when physicians are not on service in the ICU, in addition to measuring well-being when on service. As the investigators hypothesized, an ebb and flow to burnout exists, with burnout peaking at 41% when on service and subsiding to 25% when not attending in the ICU [18]. Burnout varied by rotation, implying role, staffing and ICU culture can impact burnout measures, and, as detailed below, rotation length. Furthermore, in contrast to the ebb and flow of burnout, fulfillment was common whether the physician was off service, at 60%, or during service, at 55% [18]. As context, fulfillment was observed in 34% of physicians in the validation study of the survey instrument [21]. These data suggest that fulfillment, or joy, is common in the profession of critical care medicine. Confirmatory studies engaging the entire multidisciplinary care team are warranted, as are studies designed to elucidate factors associated with professional fulfillment in the field of critical care medicine.

### The Impact of ICU Burnout Syndrome

The effects of clinician burnout syndrome are far reaching. In addition to adversely affecting the well-being of individual clinicians, burnout syndrome can have major adverse consequences for patient-care and the healthcare system [3, 4, 10].

### Individual Impact

Burnout syndrome can have a significant impact on the health and well-being of individual clinicians. For example, symptoms of depression and post-traumatic stress disorder (PTSD) are more common in ICU physicians and nurses with burnout syndrome [12, 22, 23]. This can have wide ranging effects on the individual’s private life as well as patient safety. In a recent prospective, observational multicenter study of over 1500 staff from 31 ICUs in France, symptoms of depression in healthcare staff were an independent risk factor for medical errors [24]. There is also a negative relationship between individual clinician productivity and burnout. This definition of productivity included an increased number of sick days, intent to continue practicing and intent to change jobs [25]. One study from Europe also demonstrated that physicians with burnout had significantly greater odds of having self-perceived “insufficient” work ability [26]. This lack of confidence in one’s ability may have an impact on both the practitioner’s mental health and indeed ongoing patient care.

### Healthcare System and Patient Safety

International research has demonstrated the relationship between patient-reported experience and staff burnout. For example, in one US-based study of more than 800 nurses and 600 patients from over 20 hospitals, nurse burnout was associated with patient satisfaction. In this particular study, patients who were cared for on units that nurses characterized as having adequate staff, good administrative support for care and good relations between staff groups were more than twice as likely as other patients to report high levels of satisfaction with the care they had received, and the nurses in these units reported significantly lower levels of burnout [27].

Burnout syndrome can have a bi-directional relationship with patient safety. Errors in the clinical setting can cause stress for the individual clinician involved and lead to burnout syndrome. Conversely, burnout syndrome may cause stress, reduce performance, and thus cause more errors [3, 4]. Burnout syndrome can also result in high sickness rates and potential skill drain in organizations if staff members feel they have no option but to leave their jobs prematurely to preserve their own mental and physical health; this may cause problems for the healthcare system, the individual, and also patient safety [28, 29]. Although an easy solution may appear to be to replace staff in these roles, this may not be a straightforward process and may be associated with a reduction in efficiency [29]. A recent estimate suggests that the average costs to replace an ICU nurse in the United States range from $36,657 to $88,000; thus higher turnover can have a significant economic impact for healthcare systems [30]. In more extreme cases, there may be no other suitably qualified candidates to perform the task, which may be a further risk to patient safety [31].

### Risk Factors for Burnout Among ICU Clinicians

Previous surveys of broad populations of physicians and nurses, and of critical care physicians, nurses, nurse assistants, and respiratory therapists specifically, have elucidated several factors associated with stress and burnout, which can be categorized into four broad domains: (1) individual characteristics, (2) workload and organizational issues, (3) quality of working relationships, and (4) clinical care requirements (Fig. 1) [3].

The primary individual characteristic associated with increased risk of burnout among physicians is female sex. Women physicians had approximately a 60% increased rate of burnout compared to men in both the Physician Work Life Study, which included almost 6000 physicians across a broad range of medical specialties in the United States [32], and in a survey of almost 1000 French intensivists [17]. Among nurses, female sex has not been consistently associated with the presence of burnout; however, nursing surveys have reported very high percentages of female respondents, perhaps limiting the possibility of testing this association.

Younger age has been associated with burnout among ICU nurses [14, 33]. This may reflect increased perceived stress related to inexperience or self-confidence, or that those nurses who experienced burnout left the specialty or clinical practice altogether at a younger age. Certain personality characteristics may also influence the experience of burnout. For example, among a group of nurses in Spain, neuroticism (as measured by a validated personality inventory) was associated with increased emotional exhaustion, depersonalization, and decreased personal accomplishment. Conversely, extroversion and agreeableness were potentially protective, as they were associated with decreased burnout scores [34].

A number of workload measures and organizational factors have been linked to increased burnout. Notably, the sheer volume of work (as measured by working hours) has not been demonstrated to have a consistent association; however, timing of work has. For example, among ICU nurses, lack of control over one’s schedule and rapid patient turnover is associated with increased burnout [14]. On the other hand, having professional activities outside of bedside care, such as involvement in a work group or research team, may be protective against burnout [14, 35, 36]. Among physicians, having more night shifts, more consecutive work days, and less time since the last nonworking week contribute to burnout [17, 18]. Furthermore, ICU physicians who display evidence of psychological distress or depression perceive feeling too much responsibility as a major stressor, suggesting that concurrent and competing clinical demands contribute to burnout [37].

Among physicians and nurses, working relationships have been consistently described as important contributors to job satisfaction. Numerous surveys have demonstrated an association of interpersonal conflicts—between nurses and physicians, with peers and colleagues, with supervisors, and with patients and families— with increased risk of burnout. Interpersonal conflict in the care of critically ill patients can lead to moral distress (that is, the inability of a clinician to act according to his/her values due to internal and external constraints), which has specifically been linked to burnout [38, 39]. Even in the absence of conflicts, higher scores for quality of relationships with nurses as reported by physicians were associated with less burnout [17], suggesting the importance of healthy and positive collaboration as a mechanism to protect clinicians.

Finally, the clinical care that is required of ICU clinicians may contribute to burnout. Taking care of critically ill patients is by nature stressful, fast-paced, and potentially chaotic. Although studies have not shown a consistent independent association between patient severity of illness and risk of burnout, a few studies among ICU nurses have demonstrated higher rates of burnout when caring for dying patients and being involved in decisions about withholding and withdrawing lifesustaining therapies [14, 36].

### Strategies to Mitigate Burnout

The prevalence of burnout among ICU clinicians and its potential consequences warrant immediate action. Given the breadth of risks to clinicians, to quality of care and patient outcomes of today, and to the quality and size of the ICU workforce of tomorrow, we believe that clinicians, hospital administrators, and policy makers must share in the responsibility for taking action.

Unfortunately, there has been limited empirical research thus far to guide us. To our knowledge, there have been no randomized trials of interventions focused on prevention or treatment of burnout in ICU clinicians. We suggest that candidate interventions—focused on recognition of burnout as a common syndrome in ICU clinicians, establishing and maintaining healthy collaborative work environments, and providing flexibility and resources to support clinicians experiencing burnout—should be developed and tested with the same rigor as patient-targeted therapeutic interventions in critical care.

According to the Critical Care Societies Collaborative, in addition to organizational accountability, clinicians should have “individual accountability for maintaining their own emotional and physical health and for building resiliency” [3]. To do so, clinicians must first learn how to identify burnout symptoms in themselves and their colleagues. Then, they must develop healthy strategies to ensure self-care and mitigate fatigue (such as getting adequate sleep, exercise [40], or engaging in mindfulness and meditation practices); for time management; and to optimize integration and balance between personal and professional responsibilities, all of which promote resiliency and may reduce burnout.

Clinicians should also be mindful to avoid unhealthy behaviors that can exacerbate fatigue (e.g., limit alcohol [40]), undermine health and fuel burnout. For example, 41%, 23%, and 19% of physicians in a US survey acknowledged coping with burnout by isolating themselves from others, drinking alcohol, and binge eating, respectively [16]. Rather than distancing oneself from others and disengaging, evidence suggests that engagement and a commitment to deliver compassionate care mitigates burnout, in addition to improving patient outcomes [41].

There are several ways in which organizations can address ICU clinician burnout. In general, these strategies are designed to address one or more of the “individual-to-job mismatches” that contribute to burnout: workload, control, reward, community, fairness, and values [9]. First, to prevent burnout, organizations should prioritize the creation and maintenance of healthy work environments. For example, incorporating team-building and communication training into professional development activities could improve working relationships and conflict management. Use of team debriefings after high-stress team interactions, such as cardiac arrest, can similarly promote increased and improved interpersonal communication and effective collaboration while acknowledging and applauding the team’s valuable efforts. Structured communication, such as during interprofessional rounds, can support role clarity and teamwork. Collaborative decision-making and ethical deliberation on critical decisions can also improve the ICU environment and potential mitigate moral distress.

Second, organizations can take steps to address the issues around workload and timing. Providing clinicians with some flexibility and autonomy in scheduling may provide a sense of control that promotes job satisfaction. Furthermore, putting limits on continuous working days may lessen the emotional and physical exhaustion and sleep deprivation that accompanies the high-intensity clinical care. Indeed, studies have demonstrated that changing intensivist rotations from 14 consecutive days to either 7 consecutive days or giving the weekend off in the middle is associated with reduced burnout symptoms [18, 42].

A novel strategy for the prevention of burnout syndrome is the adoption of activities that recognize the long-term recovery trajectory of patients and caregivers following the initial ICU exposure [43]. Recent multicenter work undertaken by the Society of Critical Care Medicine’s THRIVE initiative has demonstrated that longitudinal feedback improved staff satisfaction at work, as well as potentially improving patient care in the ICU [44]. This feedback can be obtained through a number of forums including peer support groups, ICU follow-up clinics, and patient and staff celebration events. This novel mechanism is still developing, and more research is required around its relationship with clinician burnout syndrome.

Finally, providing training and resources to build resiliency could improve the ability of ICU clinicians to cope with the stressful ICU environment. For example, in a recent pilot study, ICU nurses participated in a 2-day resilience training workshop on topics such as self-care, mindfulness exercises, and expressive writing therapy. Participants found this workshop acceptable and had decreased PTSD symptom scores afterwards [45]. In another pilot study of physicians, a professional coaching program reduced emotional exhaustion and improved overall quality of life and resiliency [46]. Other resources that organizations could provide include access to cognitive-behavioral therapy, establishment of support groups, and stress-reduction training.

## Conclusion

Burnout is a threat to the profession of critical care medicine, with high prevalence rates across critical care provider disciplines. However, with a robust community response to the call to action, the opportunity exists to mitigate burnout and optimize fulfillment among critical care professionals to ensure that caring, compassionate, high-quality critical care is delivered to all critically ill patients.

## Data Availability

Not applicable.
